# Neglected acromioclavicular joint injury in coracoid process fractures: a retrospective cohort study

**DOI:** 10.1007/s00402-026-06356-7

**Published:** 2026-05-27

**Authors:** Myung-Sub Lee, Chang-Hun Lee, Young-Hoon Jo, Won-Tae Cho, Young Wook Seo, Wan-Sun Choi

**Affiliations:** 1 Department of Orthopaedic Surgery, Ajou University School of Medicine, Suwon, Republic of Korea; 2 Department of Orthopaedic Surgery, Hanyang University College of Medicine, Seoul, Republic of Korea

**Keywords:** Coracoid process fracture, Superior suspensory shoulder complex, Acromioclavicular joint injury

## Abstract

**Introduction:**

Coracoid process fractures are frequently associated with injuries to the superior suspensory shoulder complex, including the acromioclavicular (AC) joint. However, concomitant AC joint injuries may be overlooked in the acute phase, potentially limiting treatment options. This study aimed to investigate the association between coracoid process fractures and SSSC injuries and to identify imaging-based predictors of concomitant AC joint injury.

**Methods:**

A retrospective single-center cohort study including 60 patients with coracoid process fractures was conducted between February 2016 and April 2023. Coracoid fractures were classified based on anatomical location, and associated SSSC injuries were categorized according to the involved structures. These injuries were evaluated using 3D shoulder CT. Morphological deformity of the SSSC was assessed by measuring the glenoclavicular distance (GCD)—a longitudinal parameter—on final follow-up clavicle AP radiographs. In base fractures, the medial displacement gap was measured on sagittal CT. Statistical analyses included logistic regression and ROC curve analysis to identify predictors of AC joint injury.

**Results:**

Among the 60 patients, fractures were located anteriorly in 27 cases, in the middle region in 5 cases, and at the base in 28 cases. AC joint injury was identified in 15 patients, all of whom had base-type fractures. Logistic regression analysis revealed a significant association between base fractures and SSSC injuries (OR = 21.0, *p* < 0.001). The GCD was significantly different between affected and contralateral sides in base fracture cases (Kruskal–Wallis test, *p* < 0.001). Receiver operating characteristic (ROC) curve analysis indicated that a displacement gap of ≥ 5.82 mm was predictive of concurrent AC joint injury (AUC = 0.741).

**Conclusions:**

Coracoid process base fractures are significantly associated with injuries to other components of the SSSC. In particular, a displacement gap of 5.82 mm or more suggests a higher likelihood of previously unrecognized AC joint injury.

**Level of evidence:**

III, retrospective cohort study.

## Introduction

Coracoid process fractures are relatively uncommon injuries, accounting for approximately 3–13% of all scapular fractures [17]. These fractures can be classified according to anatomical location or established systems such as those proposed by Ogawa and Eyres [7, 18]. They are typically associated with high-energy trauma and may occur in conjunction with injuries to surrounding structures [4]. The superior suspensory shoulder complex (SSSC) represents a ring-like structure composed of both osseous and ligamentous components, including the coracoid process, and disruption of multiple elements may result in instability and altered shoulder biomechanics. In this context, coracoid process fractures are often associated with injuries involving other components of the SSSC [10, 12, 20]. Notably, concomitant acromioclavicular (AC) joint injuries have been reported in up to 60% of such cases [14, 18].

In clinical practice, concomitant AC joint injuries may be overlooked in the acute setting, especially when radiographic findings are subtle or masked. Delayed recognition of these injuries may limit treatment options and adversely affect outcomes. Therefore, identifying reliable indicators of associated injuries is important for appropriate management. Previous studies have primarily reported treatment outcomes in patients with coracoid process fractures in whom concomitant AC joint injuries were already identified, mostly in the form of case reports or small case series [15, 28]. As such, these studies provide limited insight into the detection of initially unrecognized AC joint injuries and their implications for clinical decision-making.

The purpose of this study was to investigate the association between coracoid process fractures and injuries of the SSSC and to identify imaging-based predictors of concomitant AC joint injury. We hypothesized that specific fracture patterns, particularly base fractures with displacement, would be associated with a higher risk of AC joint injury.

## Materials and methods

**Fig. 1:**
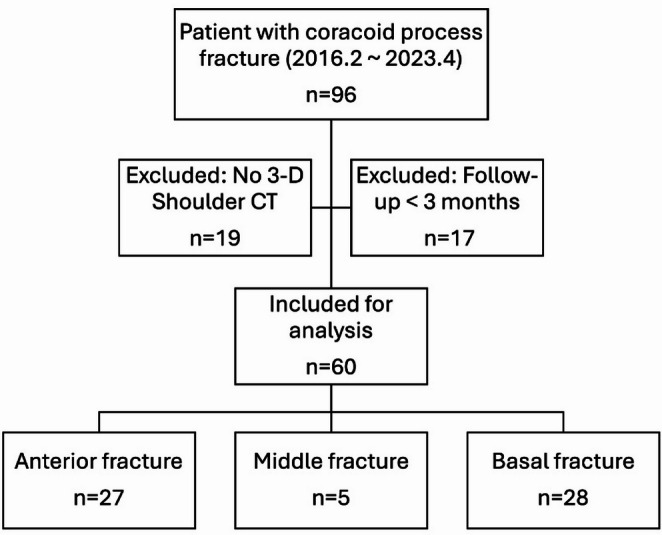
Flowchart of patient inclusion and coracoid process fracture classification

We identified patients with coracoid process fractures treated at a single institution between February 2016 and April 2023. Patients were extracted from the institutional database using the International Classification of Diseases, 10th Revision (ICD-10) code for coracoid process fracture (S42.13). Among these, only patients who underwent an initial 3-D shoulder CT scan and had at least 12 weeks of follow-up were included. Of the 96 identified patients, 36 were excluded due to lack of CT imaging or insufficient follow-up, leaving a final study cohort of 60 patients (Fig. 1).

Patient demographics, including age, sex, and surgical history, were collected through chart review. Coracoid process fractures were classified based on the initial 3-D shoulder CT. Fracture locations were categorized as anterior, middle, or base according to anatomical location. In addition, established classification systems, including the Ogawa and Eyres classifications, were applied. In this study, Ogawa type I and Eyres types III–V were considered to represent base fractures [7, 18]. Associated injuries to other components of the SSSC were identified and categorized according to the involved anatomical structures, including the acromioclavicular joint, distal clavicle, acromion, and coracoid process. The AC joint was assessed using the Rockwood classification [9]. Associations between coracoid process fracture types and concomitant SSSC injuries were analyzed using regression analysis.

**Fig. 2:**
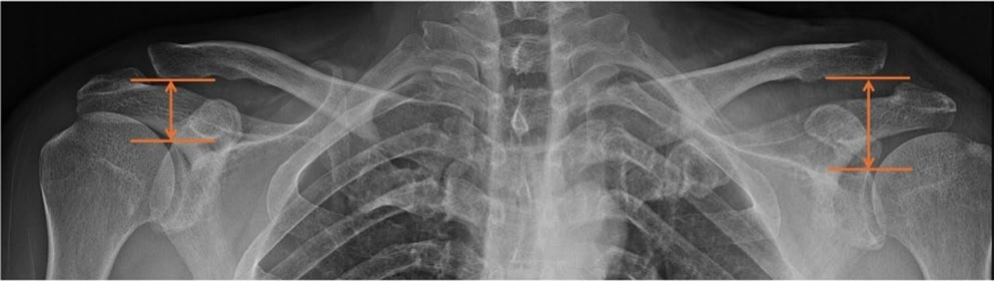
Measurement of glenoclavicular distance

At the final follow-up, simple anteroposterior (AP) radiographs of the clavicle were used to evaluate AC joint alignment and vertical deformity of the SSSC. All radiographs were obtained according to a standardized institutional protocol, with patients in an upright standing position and both upper extremities in a neutral position to minimize variation in shoulder girdle alignment. Care was taken to maintain consistent patient positioning and beam orientation across examinations. The longitudinal length of the SSSC was measured as the distance from the superior border of the glenoid rim (the base of the SSSC) to the inferior surface of the distal clavicle (the roof of the SSSC), referred to as the glenoclavicular distance (GCD). (Fig. 2) The GCD was compared bilaterally between the injured and uninjured sides. Two independent observers—a consultant upper extremity surgeon and a hand surgery fellow—measured the GCD on the AP radiographs. The fellow re-measured all radiographs one month later. Intra- and inter-observer reliability were assessed using the intraclass correlation coefficient (ICC) [22]. The average of the two observers’ GCD measurements was used for further analysis.

**Fig. 3:**
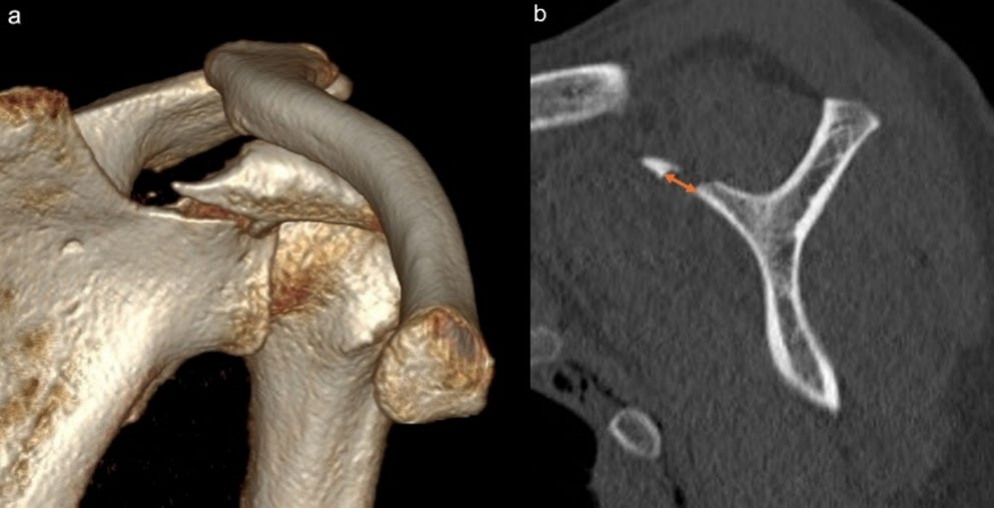
**a** An anteroinferolaterally displaced distal fragment in a coracoid process base fracture. **b** Measurement of the displacement gap at the medial edge of the fracture on a sagittal 3-D CT image

In coracoid process base fractures, the distal fragment is typically displaced anteroinferolaterally due to the pull of the biceps brachii, coracobrachialis, and pectoralis minor tendons [1, 7, 8]. This typically results in the largest fracture gap occurring along the medial margin of the fragment. The magnitude of initial displacement was assessed by measuring this gap on sagittal plane views of 3-D CT images.(Fig. 3) ICCs were also calculated for this displacement gap. Two observers independently measured the gap on the reconstructed CT slice they mutually agreed represented the most medial fracture margin. To assess intra-observer reliability, the hand surgery fellow repeated the measurements at two different time points: at the beginning of the study and one month later. Based on these measurements, receiver operating characteristic (ROC) curve analysis was performed to determine the extent of initial displacement in coracoid process base fractures that may be predictive of concomitant AC joint injury.

Result.

The study included a total of 60 patients with a mean age of 50 years (range, 25–76 years) and a mean follow-up duration of 11.9 months (range, 3–39 months). There were 44 male and 16 female patients. Fracture locations included 27 anterior, 5 middle, and 28 base fractures of the coracoid process.

Associated injuries to the SSSC included distal clavicle fractures, AC joint injuries, and acromion fractures. Distal clavicle fractures were observed in four cases (two anterior coracoid process, two basal coracoid process fractures), and acromion fractures were observed in ten cases (two anterior coracoid process, eight basal coracoid process fractures). AC joint injuries classified as Rockwood classification II or higher were initially identified in three cases. During follow-up, an additional 12 cases were diagnosed, bringing the total to 15 cases: eight classified as type II, six as type III, and one as type V injury. Notably, all cases of AC joint injury occurred exclusively in patients with coracoid process base fractures. Logistic regression analysis revealed that base fractures of the coracoid process were significantly associated with concomitant SSSC injuries. Patients with base fractures had 21 times higher odds of sustaining an SSSC injury compared to those with anterior or middle fractures (OR = 21.00, 95% CI: 5.43–81.21, *p* < 0.001). (Table 1) When each associated injury was analyzed separately, base-type fractures were also significantly associated with acromion fractures (OR, 6.00; 95% CI, 1.15–31.23; *p* = 0.033), whereas no significant association was found with distal clavicle fractures (OR, 1.15; 95% CI, 0.15–8.78; *p* = 0.890). Because all AC joint injuries occurred exclusively in the base fracture group, complete separation was observed, precluding reliable estimation of the odds ratio; however, this finding indicates a strong and clinically meaningful association.

**Table 1:** Associated injuries of the superior suspensory shoulder complex in coracoid process fractures

		Location of coracoid fracture		
		Anterior	Middle	Base
Types		Ogawa II	Ogawa II	Ogawa I
		Eyres I	Eyres II	Eyres III, IV, V
Number of patients		27	5	28
Associated SSSC^a^ injuries	Distal clavicle fracture	2	0	2
	Acromion fracture	2	0	8
	AC^b^ joint injury	0	0	15 (II:8, III:6, V:1)^c^
Logistic regression analysis for association with SCCC injury (base vs. non-base fracture)		Odds ratio = 21.00, 95%CI^d^:5.43–81.21, *p* < 0.001		

^a^Superior suspensory shoulder complex

^b^Acromioclavicula

^c^Rockwood classification

^d^Confindence interval

**Fig. 4:**
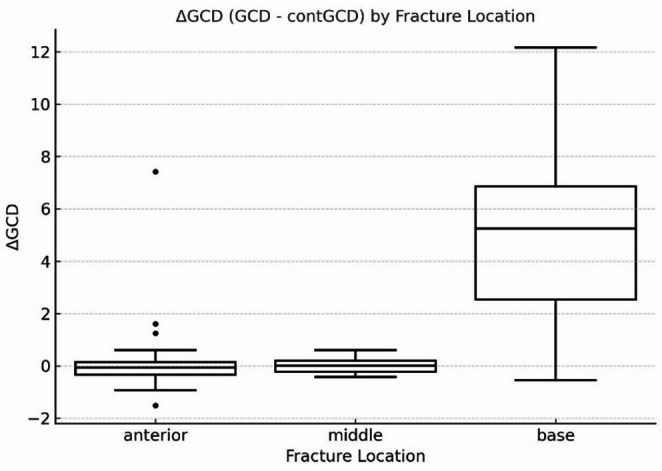
Boxplot showing the differences between glenoclavicular distance (GCD) and contralateral GCD (ΔGCD = GCD – contralateral GCD) across fracture locations. The fracture locations were categorized as anterior, middle, and base. The base group showed significantly greater ΔGCD values compared to the anterior and middle groups (*p* < 0.001, Kruskal-Wallis test)

In patients with coracoid base fractures, the GCD on the injured side was significantly greater than that on the contralateral side. A Kruskal–Wallis test demonstrated a significantly greater ΔGCD (injured GCD − contralateral GCD) in the base fracture group compared to other fracture locations (*p* < 0.001), suggesting that this fracture type is associated with more substantial structural alteration of the SSSC. (Fig. 4) The ICCs for GCD measurements were 0.802 for intra-observer reliability and 0.835 for inter-observer reliability, indicating excellent reliability [22]. 

**Fig. 5:**
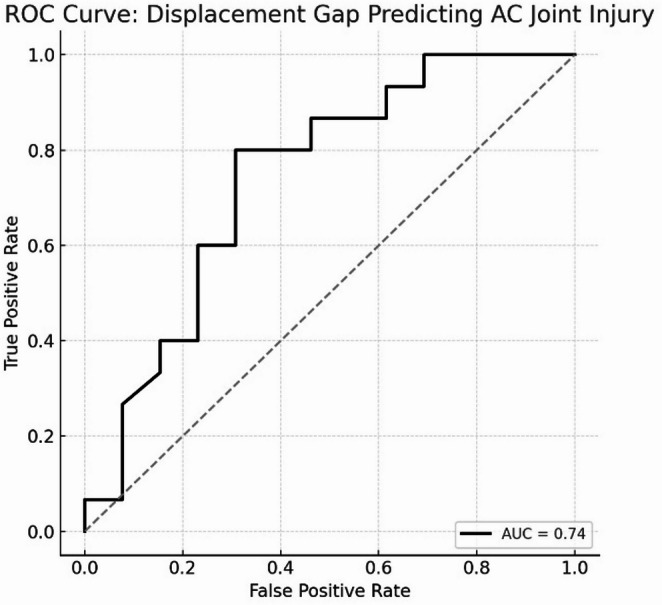
Receiver operating characteristic (ROC) curve illustrating the ability of displacement gap to predict AC joint injury. The optimal cut-off value was 5.82 mm, with a sensitivity of 0.80 and specificity of 0.69 (AUC = 0.741)

ROC curve analysis demonstrated that the displacement gap at the medial edge of the fracture had fair discriminatory power for predicting AC joint injury, with an area under the curve (AUC) of 0.741. The optimal cut-off value was 5.82 mm, yielding a sensitivity of 80% and specificity of 69%. (Fig. 5) The ICCs for displacement gap measurement were 0.831 for intra-observer and 0.789 for inter-observer reliability, both indicating excellent reliability.

**Fig. 6:**
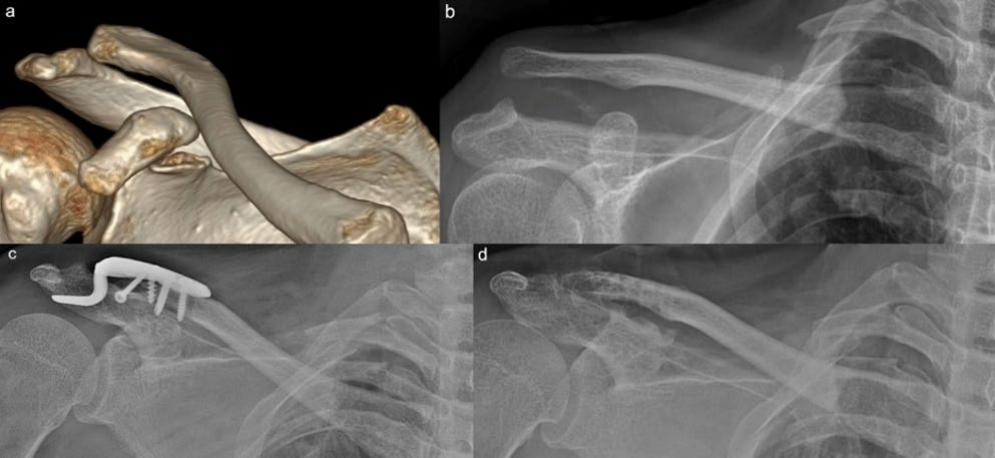
**a** Three-dimensional shoulder CT showing a displaced coracoid process fracture. **b** A Rockwood type V acromioclavicular (AC) joint injury was diagnosed at a delayed stage. **c** At 7 weeks post-injury, a modified Weaver-Dunn procedure was performed. **d** At the final follow-up, the AC joint was well reduced

**Fig. 7:**
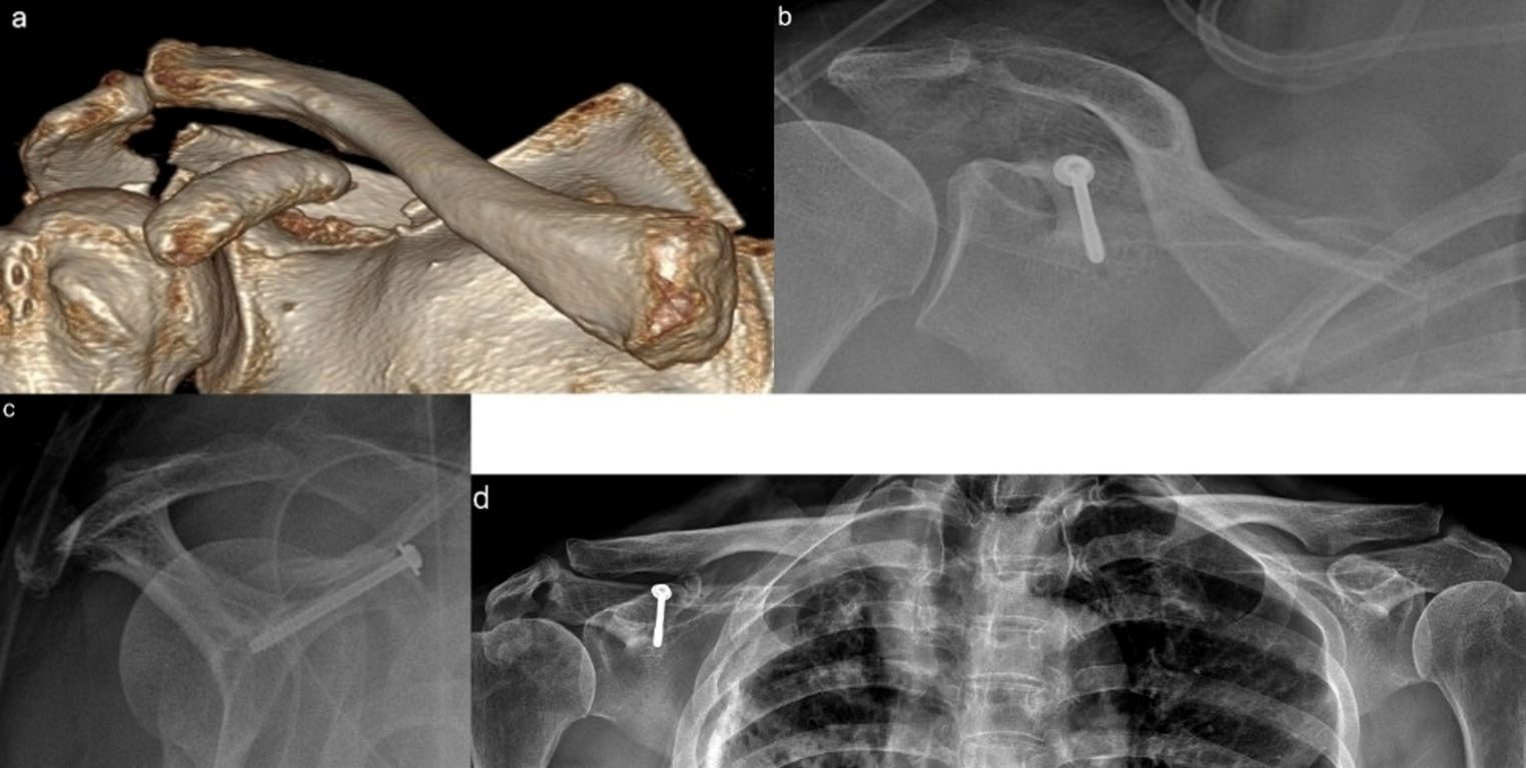
**a** Displaced coracoid process fracture with an accompanying acromion fracture, without AC joint dislocation. **b** Open reduction and internal fixation using a cannulated screw was performed. **c** Postoperative scapular outlet view showing proper screw placement and good fracture reduction. **d** At final follow-up, the longitudinal length of the superior shoulder suspensory complex was well maintained compared with the contralateral side

During follow-up, a total of seven patients were identified with Rockwood type III or higher AC joint injuries. Among them, six were diagnosed later, while one was identified at the initial assessment but could not undergo surgery due to severe brain injury. Ultimately, three of these patients underwent surgical treatment. One patient underwent open reduction and internal fixation (ORIF) for a coracoid process base fracture and AC joint dislocation at 6 weeks post-injury. The other two patients underwent a modified Weaver-Dunn procedure at 8 and 23 weeks post-injury, respectively. (Fig. 6) Among the conservatively treated cases, one patient had severe brain injury, another had multiple vertebral fractures, and two others declined surgery based on personal preference. In addition, one patient who initially showed no evidence of AC joint injury but had a 6.7 mm displacement of the coracoid base fracture underwent ORIF. At the final follow-up, the GCD on the injured side was well preserved and comparable to that of the uninjured side. (Fig. 7)

## Discussion

The clinical significance of coracoid process fractures varies depending on the fracture location. In particular, base fractures are more closely associated with injuries to the SSSC than non-base fractures. In the present study, coracoid base fractures were found to be associated with longitudinal elongation of the SSSC, and a displacement gap of ≥ 5.82 mm at the medial edge of the fracture was indicative of a higher likelihood of concomitant AC joint injury.

Previous studies have reported an association between coracoid process fractures and concomitant injuries of the SSSC, including AC joint dislocation; however, most of these studies were limited to case reports or small case series focusing on cases in which both injuries were clearly identified at presentation [2, 6, 13, 15, 28]. While these reports provided insight into treatment strategies for combined injuries [19, 26], they offer limited information regarding the detection of initially unrecognized AC joint injuries. In contrast, the present study demonstrates that AC joint injuries may be initially overlooked and are strongly associated with specific fracture patterns, particularly base fractures with displacement. This difference may be explained by the inclusion of patients with subtle or initially missed injuries in our cohort. Therefore, our findings highlight the importance of recognizing coracoid base fractures as a potential indicator of occult AC joint injury.

In clinical practice, concomitant AC joint injuries may be overlooked in the acute setting, especially when radiographic findings are subtle or masked. Delayed recognition of these injuries may limit treatment options and adversely affect outcomes. AC joint dislocations identified early can often be effectively treated with open reduction and internal fixation (ORIF) [23, 25]. In contrast, delayed diagnoses may require more complex surgical procedures such as ligament reconstruction or ligament transfer (e.g., Weaver-Dunn procedure) [5, 21, 24]. While conservative treatment may still be an option depending on the patient’s symptoms and functional demands, once surgery is indicated, the complexity of the procedure increases the risk of complications and surgical failure [3, 11, 16, 27]. Therefore, early diagnosis and treatment are preferable. A displaced coracoid base fracture strongly suggests the potential presence of an unrecognized AC joint injury and may help clinicians avoid missing the optimal window for acute-phase intervention.

These findings may, in part, be explained by the limitations of standard radiographic evaluation in the acute trauma setting. Although standard radiographs are generally considered sufficient for detecting obvious AC joint injuries, concomitant injuries may be underdiagnosed. Radiographs are often obtained in supine or supported sitting positions in severely injured patients, which may reduce physiological stress across the AC joint. Consequently, joint instability may not be adequately demonstrated, particularly in cases of partial ligament injury or spontaneously reduced dislocation. In this context, the presence of a displaced coracoid process fracture should raise suspicion for a concomitant AC joint injury, even when radiographic findings appear inconclusive. Recognizing this association may help clinicians avoid missed diagnoses and facilitate timely and appropriate management.

This study has several limitations. First, functional outcomes were not evaluated, and radiological healing was not systematically assessed. However, as the primary aim of this study was to identify imaging-based predictors of concomitant AC joint injury rather than to evaluate treatment outcomes, outcome analysis was beyond the scope of this study. Second, because this was a retrospective cohort study, uniform follow-up imaging and treatment protocols could not be applied to all patients. However, all patients were managed at a single institution, which may have contributed to a relatively consistent treatment approach. Third, we were unable to compare clinical outcomes based on treatment protocols. The follow-up periods varied, and especially for conservatively treated patients, the duration was relatively short, making it difficult to directly compare outcomes within a standardized time frame. Future studies incorporating standardized follow-up protocols and clinical outcome assessments are warranted to further validate the clinical implications of our findings.

In conclusion, coracoid process base fractures were significantly associated with injuries to other components of the superior suspensory shoulder complex (SSSC). In particular, a displacement gap of 5.82 mm or more at the medial edge of the fracture was associated with a higher likelihood of concomitant, initially unrecognized acromioclavicular (AC) joint injury. As AC joint dislocations are more easily treated in the acute phase than in chronic cases, careful evaluation for associated injuries and timely consideration of appropriate management are warranted in patients with displaced coracoid base fractures.

## Data Availability

The data that support the findings of this study are not publicly available due to ethical and privacy restrictions but are available from the corresponding author upon reasonable request.
